# A snail-eating snake recognizes prey handedness

**DOI:** 10.1038/srep23832

**Published:** 2016-04-05

**Authors:** Patchara Danaisawadi, Takahiro Asami, Hidetoshi Ota, Chirasak Sutcharit, Somsak Panha

**Affiliations:** 1 Biological Science Program, Department of Biology, Faculty of Science, Chulalongkorn University, Bangkok 10330, Thailand; 2 Animal Systematics Research Unit, Department of Biology, Chulalongkorn University, Bangkok 10330, Thailand; 3 Department of Biology, Shinshu University, Matsumoto 390-8621, Japan; 4 Institute of Natural and Environmental Sciences, University of Hyogo, and Museum of Nature and Human Activities, Hyogo 669-1546, Japan

## Abstract

Specialized predator-prey interactions can be a driving force for their coevolution. Southeast Asian snail-eating snakes (*Pareas*) have more teeth on the right mandible and specialize in predation on the clockwise-coiled (dextral) majority in shelled snails by soft-body extraction. Snails have countered the snakes’ dextral-predation by recurrent coil reversal, which generates diverse counterclockwise-coiled (sinistral) prey where *Pareas* snakes live. However, whether the snake predator in turn evolves any response to prey reversal is unknown. We show that *Pareas carinatus* living with abundant sinistrals avoids approaching or striking at a sinistral that is more difficult and costly to handle than a dextral. Whenever it strikes, however, the snake succeeds in predation by handling dextral and sinistral prey in reverse. In contrast, *P. iwasakii* with little access to sinistrals on small peripheral islands attempts and frequently misses capturing a given sinistral. Prey-handedness recognition should be advantageous for right-handed snail-eating snakes where frequently encountering sinistrals. Under dextral-predation by *Pareas* snakes, adaptive fixation of a prey population for a reversal gene instantaneously generates a sinistral species because interchiral mating is rarely possible. The novel warning, instead of sheltering, effect of sinistrality benefitting both predators and prey could further accelerate single-gene ecological speciation by left-right reversal.

Left-right asymmetry has repeatedly evolved in the external morphology and behavior of many bilaterian animals which are symmetric in body plan^1^,^2^,^3^,^4^,^5^,^6^,^7^. This secondary asymmetry often plays crucial roles in interactions with the external environment, in contrast to internal asymmetry which results from primary asymmetry expressed in early development^8^,^9^. Directional asymmetry in the feeding apparatus of snail-eating specialists may represent a direct role of secondary asymmetry in predator-prey interactions which drive dynamics of ecology and evolution in living communities^5^,^10^,^11^,^12^,^13^,^14^,^15^.

Southeast Asian snakes of the genus *Pareas* typically specialize in the predation of snails by extracting the soft body from the shell^16^,^17^ and exhibit directional asymmetry in mandibular dentition^5^. *Pareas iwasakii* strikes at prey by tilting the head leftward only and attempts to capture by the same handling manner irrespective of the direction of snail coil (handedness)^5^, which can either be clockwise (dextral) or counterclockwise (sinistral) (Fig. 1). Because of the lack of recognition of prey handedness, this snake frequently fails in capturing the sinistral prey with its mouthparts, and thus this prey survives. Poor performance of predation on sinistral prey (sinistral-predation) suggests that the asymmetries of morphology and behavior of *Pareas* snakes evolved for specialized predation on the dextral majority in snails and thus do not effectively function for sinistral-predation^5^,^15^.

In sympatry with *Pareas* snakes, most snails are pulmonates, in which a single gene reverses the direction of primary and secondary asymmetries^8^,^18^. Because of these reversals, the genital position and mating behaviors mismatch between dextral and sinistral snails. This interferes with their copulation and results in positive frequency-dependent selection against reversal^19^,^20^. However, once the reversed phenotype exceeds 50% under a survival advantage against the right-handed predation by *Pareas*, the population in isolation rapidly becomes fixed for the reversal. This instantaneous peak shift completes premating isolation, which gives rise to a reversed species^21^,^22^,^23^,^24^. Predation on dextral prey (dextral-predation) by *Pareas* snakes has contributed to the exceptionally high diversity of sinistral snails in Southeast Asia by accelerating this single-gene adaptive speciation by left-right reversal^15^.

Among *Pareas* species, however, the degree of mandibular asymmetry, which is left-right difference relative to the total number of teeth, greatly varies from 1% for a slug-eating specialist to 18% for *P. iwasakii*^5^. The latter lives on only two peripheral islands in the range of *Pareas* distribution, where only one of 23 potential prey species is sinistral (see the method for counting the potantial prey species). On the other hand, most of the other congeneric snakes occur in the central region of Southeast Asia with diverse sinistral species^15^,^25^,^26^,^27^. Such local variations in both the strength of predator asymmetry and the relative abundance of sinistral prey in sympatry may involve a geographic mosaic of coevolution^28^.

*Pareas carinatus* occurs most widely across the central region of *Pareas* distribution and exhibit the relatively weak dental asymmetry^5^,^29^ in the genus. When a relatively small snail was provided each time, this snake struck by tilting its head either leftward or rightward and succeeded in every attempt of dextral- or sinistral-predation^29^. The previous studies with *P. iwasakii* have examined the effect of prey size only by comparing the snake’s responses between large and small prey categories^5^,^15^. In the present study, we examined the effects of prey size and handedness on predatory performances of *P. carinatus* by using snails that continuously vary in shell size as widely as available in its habitat.

Here we show that a snail-eating snake *P. carinatus* refrains from behavioral attempts of costly predation on sinistral prey as predicted by the size-dependent decline of feeding efficiency in sinistral-predation relative to dextral-predation.

## Results and Discussion

### Recognition of prey handedness

In every experiment the snake moved its eyes to stare at the crawling snail as soon as the latter was placed in front of the snake. While staring, the snake approached 64 of 76 (84.2%) dextrals and 26 of 38 (68.4%) sinistrals presented. In the rest of the cases, the snake averted its head and eyes from the snail and did not approach. When approaching, the snake kept staring at the snail. During this period, the snake often reoriented its head to the snail by shifting the direction and angle of head-tilting at a distance of 10 to 20 mm from the snail. These behaviors during continuous staring suggest the importance of vision for critical operation of the mandibles and upper jaws at the subsequent moment of strike. The snake struck at 52 of the 64 (81.2%) dextrals and 19 of the 26 (73.1%) sinistrals. In the rest of the approaching cases, the snake moved away from the snail without striking.

There was no difference in shell size between approached and non-approached dextral snails (Fig. 2a). In contrast, approached sinistrals were smaller than non-approached ones. This was significant in terms of the effect of interaction between shell size and handedness on this positive or negative decision for approach (F_1,110_ = 5.4, p = 0.013, Fig. 2a). Similarly, dextrals that were struck did not differ in shell size from dextrals that were not struck, whereas sinistrals struck were significantly smaller than sinistrals not struck (F_1,86_ = 6.0, p = 0.016). The mean shell sizes presented to the snake did not differ between these dextrals and sinistrals (see materials and methods). Therefore, the snake dinstinguished prey enatiomorphs during staring and approaching.

Neither the time lengths of staring nor of approaching depended on the subsequent decisions (F_1,64_ = 1.1, p = 0.29; F_1,79_ = 1.3, p = 0.26, respectively) or on snail handedness (F_1,64_ = 0.03, p = 0.87; F_1,79_ = 0.15, p = 0.70, respectively). On average, snakes made decisions for approach in 10.3 sec ± 2.5 S.E. and for strike in 63.9 sec ± 7.9. The snake was given no choice of handedness of prey. Nevertheless, the snake frequently refrained from approaching or striking at a relatively large sinistral by staring for around 10 seconds or one minute, respectively.

Squamate reptiles use tongue-flicks for vomeronasal chemoreception^30^. In the present experiment, however, the snake did not flick the tongue in 85 of 111 (76.6%) cases before the decision for approach or in 54 of 88 (61.4%) cases before the decision for strike. In the rest (tongue-flicking cases), the number of tongue-flicks did not depend on the shell size (F_1,11_ = 0.03, p = 0.86 before the decision for approach; F_1,20_ = 0.001, p = 0.99 before the decision for strike) or on snail handedness (F_1,11_ = 0.08, p = 0.78 before the decision for approach; F_1,20_ = 3.5, p = 0.076 before the decision for strike).

Without flicking the tongue, the snake could not obtain odors for vomeronasal chemoreception. Nevertheless, the snake ceased staring at and did not approach relatively large sinistrals (F_1,81_ = 7.1, p = 0.009, Fig. 2b). After it approached in the other cases, the snake also refrained from striking at one dextral and two sinistrals without flicking the tongue. These sinistrals not struck may have been larger than the other sinistrals struck, but more replicates are necessary for statistical validation (Fig. 2b). In this predation experiment with no choice of prey handedness, the snake avoided preying on relatively large sinistrals whereas it preyed on dextrals irrespective of the shell size (F_1,110_ = 11.7, p = 0.001; Fig. 2c). This was also the case when the snake did not flick the tongue (F_1,62_ = 9.1, p = 0.004). The snake therefore recognizes prey handedness without relying on vomeronasal chemoreception by tongue-flick.

The head-tilting direction for strike varied among predation events irrespective of snake individuals (Fig. 3). This direction was rightward more frequently for sinistral prey than for dextral prey (F_1,67_ = 4.1, p = 0.046). This indicates that prey handedness affects the left-right direction of predatory behavior. In every strike the snake successfully captured and fed the prey, unlike *P. iwasakii*^5^,^15^.

While handling the prey after strike, the snake held the ventral outer surface of the last whorl (umbilicus side) with the upper jaws and the soft body with the mandibles inserted into the aperture (Fig. 4). When the prey was dextral, the left mandible was at the peripheral side of the whorl and the right mandible at the side of the shell columellar (umbilicus) (Fig. 4a). On the other hand, whenever preying on the sinistral, the snake oppositely positioned the left and right mandibles with no change of the upper jaws’ location on the ventral shell surface (Fig. 4b). This means that the snake laterally reverses the manner of prey handling according to the direction of shell asymmetry. Otherwise, in the case of sinistral prey, the snake would have directed the upper jaws toward the dorsal outer surface of the shell aperture and often fail in prey capture, as known for *P. iwasakii*^5^,^15^.

Different snail species have different odors^31^. However, our results rule out olfactory recognition of sinistral species by the vomeronasal system, which is typically important for squamates’ chemical recognition^30^. In the distribution range of *Pareas*, prey reversal frequently evolved in response to specialized dextral-predation by the snakes^15^. These phylogenetically independent sinistral lineages reversed by a single gene^15^,^24^ would be unlikely to evolve to release sinistral-specific odors if any. Of 29 sinistrals of *Dyakia salangana* given to the snake, which must have shared species-specific odors, the snake only approached 17 that were smaller in the mean shell size (14.6 mm ± 1.6 S.E.) than the rest (23.7 ± 0.87; F_1,27_ = 8.8, p = 0.006). After approach, the snake struck at the smaller 10 (11.3 ± 1.6) but not at the other larger sinistrals (22.1 ± 1.1; F_1,27_ = 8.7, p = 0.006). These size-dependent decisions for predation on conspecific prey are not ascribable to prey odor differences.

**Figure 1:**
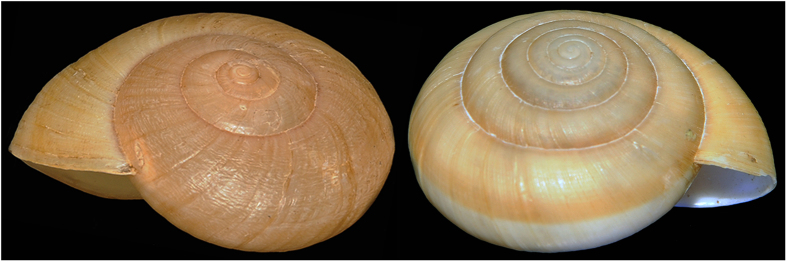
The sinistral shell of *Dyakia salangana* (left) and the dextral shell of *Cryptozona siamensis* (right).

**Figure 2:**
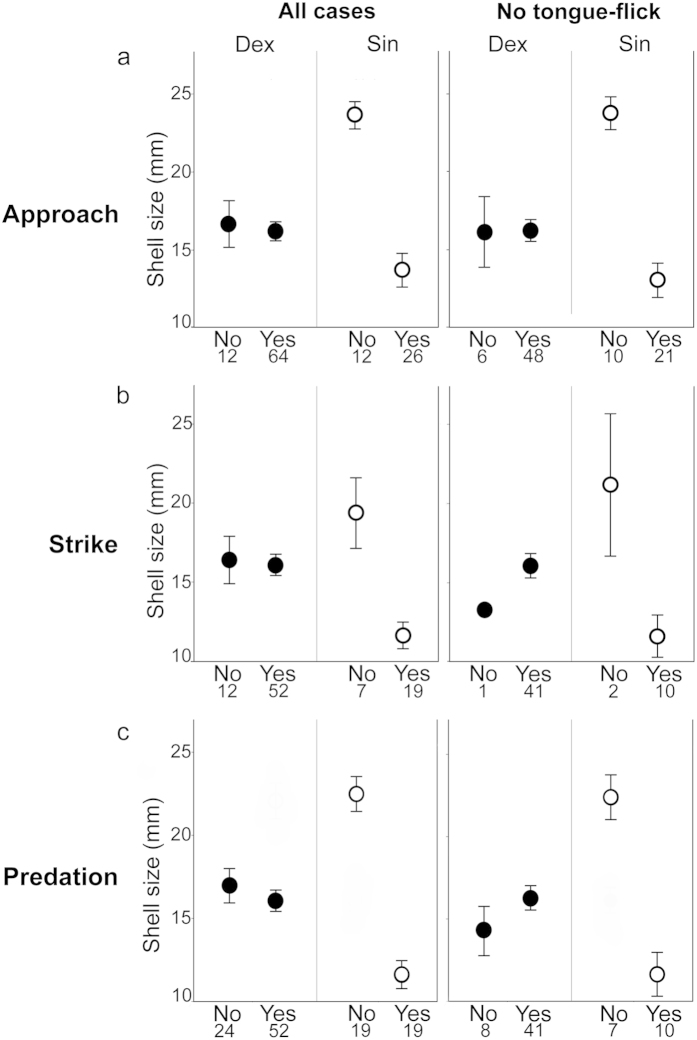
Handedness-dependent size effects on decisions for approach (**a**) and strike (**b**), and size-dependent predation on sinistrals (**c**). No and Yes are the negative and positive decisions, respectively. The snake strikes at a snail only after approach. Each number under the decision indicates the number of replicates. Each of plots and error bars indicates the mean and standard error.

**Figure 3:**
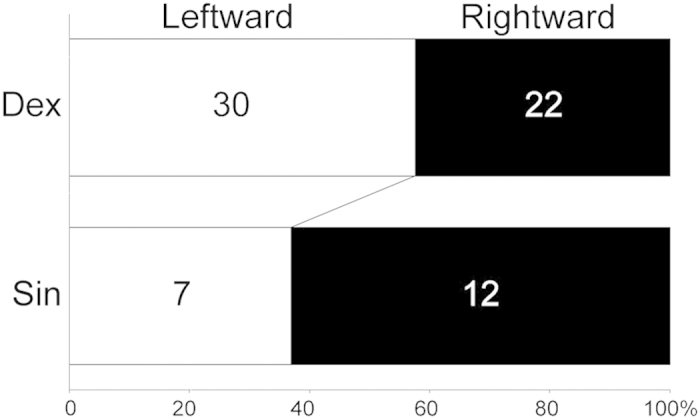
Frequencies of leftward and rightward strikes at dextral and sinistral prey. Each value indicates the number of replicates. Predation succeeded in every striking occasion.

**Figure 4:**
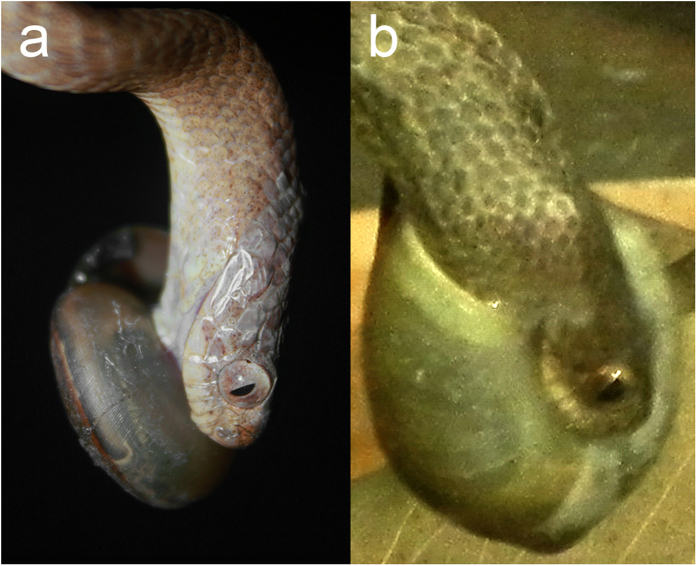
Left-right reversal of handling behavior in *Pareas carinatus* according to prey handedness. (**a)** Handling of a dextral snail (*Cryptozona siamensis*). (**b)** Handling of a sinistral snail (*Dyakia salangana*). Left and right mandibles are inserted into the aperture in reverse relative to the structure of shell whorl while the upper jaws hold the ventral outer surface (umbilicus side) of the shell.

For predation success in extracting the soft body, which is otherwise withdrawn into the shell, snail-eating snakes must locate the mouthparts properly onto the asymmetric shell at the moment of strike^5^. *Pareas carinatus* reverses such definite orientation of the apparatus by staring at sinistral prey (Fig. 4). *Pareas* snakes have a developed optical system with the large eyes for night vision as well as other nocturnal reptiles do^32^,^33^,^34^,^35^. These suggest that visual structure perception is necessary for their chirally specialized predation and overrides chemical odor distinction^36^ where prey is visible. Dextral and sinistral shells are physically discrete in coiling direction and lateral location of the aperture, through which the soft body is extracted (Fig. 1). These major differences in shell structure may serve as a visual cue for the snake to distinguish between prey enantiomorphs.

### Feeding efficiency

**Figure 5:**
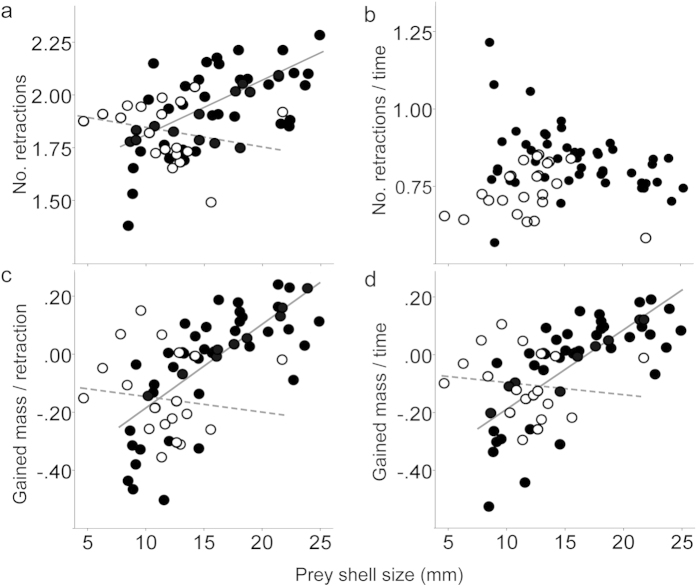
Size-dependent efficiencies and benefits of preying on a dextral. (**a**) Size dependent increase of the number of mandibular retractions only in dextral-predation. (**b**) Higher retraction frequency in dextral-predation than in sinistral-predation. (**c**) Size-dependent increase of soft-body mass gained per retraction only in dextral-predation. (**d**) Size-dependent increase of relative benefit only in dextral-predation. Time is the feeding time in seconds. Mass is the prey weight reduction in grams after predation. Solid and open circles indicate predations on dextrals and sinistrals, respectively. The regression (interrupted) line for sinistrals is illustrated in each case to indicate the intersection with that (solid line) for dextrals, though the slope for the former was not significant (see Table S1 for regression statistics).

The snake took a longer time to finish feeding on a larger snail regardless of prey handedness (F_1,64_ = 4.6, p = 0.036) and retracted the mandibles for a larger number of times while taking longer (F_1,64_ = 057, p < 0.001). However, the number of retractions increased only with the dextral prey size (F_1,47_ = 31, p < 0.001), but did not with the sinistral prey size (F_1,17_ = 0.65, p = 0.43) (Fig. 5a). Thus, difference in the number of retractions between prey enantiomorphs depended on the prey size (F_1,64_ = 9.7, p = 0.003).

The snake rectracted the mandibles more frequently while preying on the dextral than the sinistral (F_1,64_ = 5.3, p = 0.024) (Fig. 5b). The prey soft-body mass gained per retraction increased with the dextral prey size (F_1,47_ = 66, p = 0.024) but did not with the sinistral prey size (F_1,17_ = 0.008, p = 0.93) (Fig. 5c). The interaction between the size and handedness was accordingly significant (F_1,30_ = 8.1, p = 0.008). Thus, when preying on the dextral, mandibular retractions are not only more frequent but also increasingly more efficient with the prey size in terms of soft-body gain per retraction.

Superior performances in feeding on dextral prey in terms of retraction frequency and efficiency synergistically resulted in a significantly larger gain of soft body per time than that achieved by feeding on sinistral prey (F_1,30_ = 8.5, p = 0.007) (Fig. 5d). This benefit of preying on the dextral instead of the sinistral increased with the shell size (F_1,30_ = 8.0, p = 0.008), as the gain per time positively depended on the dextral’s size (F_1,47_ = 57, p = 0.001) but not on the sinistral’s size (F_1,17_ = 0.15, p = 0.70).

In Fig. 5a,c,d, regression lines for the dextral and sinistral cases cross at the shell sizes of 11.4, 12.1 and 12.7 mm, respectively (see Table S1 for the regression statistics). This predicts that the relative value of sinistral prey declines with the increase of the size. In practice, the snake preyed on all of the sinistrals smaller than 12.4 mm. However, the snake did not strike at 18 of the 26 (69.2%) sinistrals larger than this size. These cases of avoidance do not appear in Fig. 5, but nevertheless correspond to the range beyond the predicted threshold size of around 12 mm. These results support the hypothesis that the size-dependent increase of cost for preying on a sinistral instead of a dextral has driven the evolution of prey-handedness recognition and size-dependent avoidance of sinistral-predation.

In the distribution range of *P. carinatus*, sinistral species reach 17.0% in the total of 900 pulmonate species, exclusive of those that are too small for the snake to prey (see methods; Table S2). Our field records demonstrate that this arboreal snake is frequently active on trees where tree snails of pulmonates co-occur (Table S3). These tree snail species are almost invariably sinistral (subgenus *Syndromus*) or chirally dimorphic within populations (subgenus *Amphidromus*). Their high abundances are well established^26^,^27^,^37^,^38^. On the other hand, no sinistral tree snail co-occurs with a congeneric snake *P. iwasakii*, which lives on the islands with only one ground-dwelling sinistral and 22 dextral species and thus would rarely encounter sinistral prey. Thus, it would be of little advantage to evolve an ability to distinguish between prey enantiomorphs. This explains the frequent failure of *P. iwasakii* to capture a given sinistral after striking. In contrast, *P. carinatus* lives with abundant sinistral snails, where avoiding predatory attempts on costly sinistrals should be advantageous.

The predator in this case does not evolve to exploit sinistrals by arms race. Instead the snake has shifted to avoid a cost of attempting unsuccessful or inefficient sinistral-predation because the easier prey type (dextrals) still remains abundant. This behavioral response by visual recognition reduces both a risk for the snake to expend foraging time and energy to handle unsuitable prey and a risk for sinistral snails to undergo physical attacks by the snake. Sinistrality therefore functions as a warning sign to the predator, instead of sheltering the prey. Predator’s recognition of prey handedness, which benefits both the snake predator and sinistral prey, could further accelarate ecological prey speciation^39^ by a reversal gene.

Many studies have shown the association of ecological performance with the direction of asymmetry in morphology and/or behavior^4^,^5^,^11^,^20^,^40^,^41^,^42^. We do not know, however, how important it is for a predator to be so asymmetric for chirally specialized predation. The previous studies ascribed the reduced efficiencies of sinistral-predation by *P. iwasakii* to its leftward-fixed strike with no prey-handedness recognition and to its most pronounced dental asymmetry in *Pareas*^5^. In contrast, the present snake *P. carinaus* does not fail in dextral or sinistral-predation either by leftward or rightward striking. Moreover, the mean dental asymmetry among four of the six snakes used in this study was almost minimal (4.5%) in the genus^29^. Nevertheless, efficiencies of dextral-predation were obviously superior to those of sinistral-predation. Accordingly, specialized handling of asymmetric prey does not necessarily require so manifest directional asymmetry in striking behavior or mandibular morphology as expected from the previous studies.

However, *P. iwasakii* fed on the dextral wild-type of *Bradybaena similaris* more efficiently than its sinistral mutants^5^, which were as small as the present threshold size (Fig. 5). *Pareas carinatus* in contrast feeds on dextral and sinistral prey of this size range with equivalent efficiency. Thus, the efficiency of feeding on dextral prey relative to that on sinistral prey may depend on the strength of dental asymmetry. If this is the case, weaker dental asymmetry may represent weaker specialization in dextral-predation. This would be advantangeous for *Pareas* snakes where frequently coming across sinistral snails, because snails of even large species can be small enough to prey when they are young. Strongly right-handed dentition on the other hand would benefit them for strong specialization in habitats with few sinistrals. Testing this hypothesis requires explicit comparison of predation performance among snail-eating snakes that differ in dental asymmetry. Our results therefore have important implications for further investigation of functional significance of predator’s asymmetry for chirally specialized predation.

A single gene is responsbile for the reversal of primary and secondary asymmetries in pulmonate snails^8^,^18^. The snail-eating snake *P. carinatus* notices this reversal by staring at a snail, though people often do not unless told so. Our study demonstrates that a chirally specialized predator can evolve an ability to recognize the left-right reversal of prey asymmetry where advantageous.

## Materials and Methods

**Figure 6:**
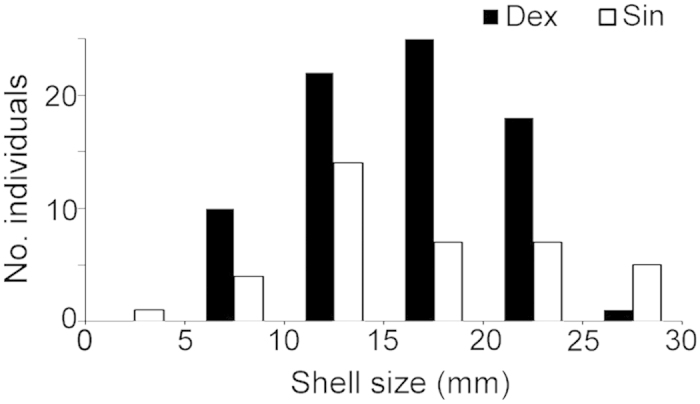
Distributions of shell size in dextral and sinistral snails used in predation experiments.

We collected six adults of *P. carinatus* (snout-vent length 510–720 mm; head width 6.78–11.53 mm) from Chanthaburi, eastern Thailand, where we also collected snails for the present study. In total, we used 76 low-spired dextral snails (56 *Cryptozona siamensis*, 2 *Ganesella capitium*, 16 *Sarika resplendens* and 2 *Satsuma* sp.) and 38 low-spired sinistral snails (29 *Dyakia salangana* and 9 *Ganesella rhombostomus*) (Table 1). To examine the effects of snail body size, we used the square root of the product of shell height by width as the shell size of each snail. These dextrals and sinistrals did not significantly differ in the mean shell size (t = −0.56, d.f. = 112, p = 0.58, Table 1, Fig. 6).

In comparison of snail faunas, we excluded the Clausiliidae and minute species with the shell which is smaller than 5 mm in diameter, height or aperture from the count of species. We treated the speciose subgenera *Amphidromus* and *Syndromus* of the genus *Amphidromus* as separate genera and counted each of the five genera, *Camaena*, *Chersaecia*, *Amphidromus*, *Ganesella*, *Syndromus*, which include both dextral and sinistral or dimorphic species, as a dextral genus as well as a sinistral genus (Table S2).

### Predation experiment

Each snake was conditioned with no food for 3 days before each predation trial (hereafter called experiment) begun at 21:00. We first placed a snail and let it crawl 100 mm ahead of a snake sitting on a horizontal wooden bar (15 mm diameter) in a transparent plastic terrarium (300 × 450 × 250 mm). We randomized the combination of snake and snail individuals. All experiments were conducted in the laboratory at 25 to 28 °C under the illuminance of 100 lux. Behavioral responses of each snake were recorded with a video camera. We conducted the experiment between October 2012 and June 2014 at Chulalongkorn University. The experimental protocol was approved by the Animal Care and Use Committee of Faculty of Science, Chulalongkorn University and carried out in accordance with the approved guidelines of the Animal Care and Use Committee of Faculty of Science, Chulalongkorn University (Protocol Review No. 1223003).

### Behavior record

The snake began to stare by fixing eyes onto the crawling snail when the latter was placed. In many cases, the snake approached the snail by moving its head and body. When the snake preys, it strikes at the snail and feeds by extracting the soft body through alternate retractions of left and right mandibles^17^,^29^. The snake drops the shell at the end of predation. By using video records, we quantified the feeding time (from striking to dropping of the shell) in seconds, the total number of retractions of left and right mandibles during feeding, and the total numbers of tongue-flicks before approach and strike. When the snake preyed, we estimated the prey soft-body mass gained by the snake by measuring a reduction of prey weight in grams after predation.

### Statistical analysis

We tested the dependence of occurrence of approach or strike on snail handedness, shell size and their interaction, by constructing generalized linear mixed models (GLMMs) with the random effects of snake individual and snail species. We logarithm-transformed the feeding time, number of retractions and prey mass gained by the snake. By using those values, we calculated the retraction frequency, prey mass gained per retraction and prey mass gained per time. We examined the fixed effects of snail handedness, shell size and their interaction on those feeding time, number of retractions, retraction frequency, and prey masses gained per retraction and per time by using GLMMs with the random effects of snake individual and snail species. We tested the fixed effects of snail handedness and size on the head-tilting direction for strike by using a GLMM with random effects of snake individual and snail species.

## Additional Information

**How to cite this article**: Danaisawadi, P. *et al.* A snail-eating snake recognizes prey handedness. *Sci. Rep.* **6**, 23832; doi: 10.1038/srep23832 (2016).

## Supplementary Material

Supplementary Information

## Figures and Tables

**Table 1:** Mean size ± S.E. of snails used in predation experiment.

Coilingdirection	Species	n	Width (W)	Height (H)	Shell size(square root of W × H)		
					Mean ± S.E.	Min	Max
Dextral	*Cryptozona siamensis*	56	13.28 ± 0.53	21.02 ± 0.95	16.67 ± 0.71	7.01	27.08
	*Ganesella capitium*	2	12.16 ± 0.08	14.53 ± 0.12	13.29 ± 0.19	13.27	13.30
	*Sarika resplendens*	16	10.31 ± 0.40	19.57 ± 0.92	14.17 ± 0.57	9.16	17.17
	*Satsuma sp.*	2	18.58 ± 0.05	26.83 ± 0.16	22.31 ± 0.04	22.28	22.35
Sinistral	*Dyakia salangana*	29	22.39 ± 1.67	15.06 ± 1.05	18.34 ± 1.30	4.65	29.96
	*G. rhombostomus*	9	11.06 ± 0.16	12.49 ± 0.52	12.02 ± 0.35	10.29	13.53
