# Supplementary material for: A snail-eating snake recognizes prey handedness
Source: Sci Rep. 2016 Apr 5;6:23832. doi: 10.1038/srep23832 (PMC4820687; doi:10.1038/srep23832)
Supplement: Supplementary Information [file srep23832-s1.pdf]

Supplementary information

Article title: A snail-eating snake recognizes prey handedness

Authors: Patchara Danaisawadi, Takahiro Asami, Hidetoshi Ota, Chirasak Sutcharit,

Somsak Panha

Table S1. Regression of each predation performance on prey size (see Fig. 5 for details).

Performance	Dextral prey		Sinistral prey	
	Slope (probability)	Interecept	Slope (probability)	Interecept
No. of retractions	0.022 ($p < 0.001$)	1.58	-0.008 ($p = 0.43$)	1.92
Gained mass/retraction	0.028 ($p = 0.024$)	-0.48	-0.004 ($p = 0.93$)	0.085
Gained mass/time	0.038 ($p = 0.001$)	-0.59	-0.004 ($p = 0.70$)	0.054

Table S2. Approximate numbers of potential-prey taxa for *Pareas carinatus*.

Family	Genera	Number of species		
		Dextral	Sinistral	Dimorphic
Camaenidae	*Camaena*	35	5	0
	Stegodera	0	1	0
	Traumatophora	1	0	0
	Trichelix	6	0	0
	Moellendorffiella	1	0	0
	Moellendorffia	4	0	0
	Neocepolis	8	0	0
	Trachia	18	0	0
	Oreobba	2	0	0
	Camaenella	1	0	0
	Burmochloritis	3	0	0
	Ganesella	49	1	0
	Globotrochus	1	0	0
	Giardia	0	3	0
	Pseudoportula	0	3	0
	Ptychochloritis	6	0	0
	Amphidromus	2	0	48
	Syndromus	1	44	1
	Eustomopsis	20	0	0
	Minacispira	3	0	0
	Trichochloritis	12	0	0
Helicarionidae	*Geotrochus*	3	0	0
	Platymma	1	0	0
Ariophantidae	*Sophina*	6	0	0
	Cryptaustenia	20	0	0
	Sivella	10	0	0
	Oxytesta	8	0	0
	Macrochlamys	120	0	0
	Porratella	9	0	0
	Sakiella	3	0	0
	Baiaplecta	1	0	0
	Naninia	10	0	0
	Koratia	1	0	0
	Hemiplecta	20	0	0
	Euplecta	30	0	0
	Ariophanta	17	0	0
	Sarika	10	0	0
	Sitala	30	0	0
	Sitalinopsis	3	0	0
	Taphrospira	4	0	0
Ryssotidae	*Exrhysota*	0	1	0
	Lamarckiella	14	0	0
Trochomorphida	*Benthemia*	2	0	0
	Eurybasis	15	0	0
	Trochositala	1	0	0
Bradybaenidae	*Nesiohelix*	9	0	0
	Landouria	15	0	0
	Thaitropis	2	0	0

	Torobaena	3	0	0
	Bradybaena	25	0	0
	Vitrinula	20	0	0
	Aegista	40	0	0
	Trichocathaica	1	0	0
	Chalepotaxis	2	0	0
Dyakiidae	*Asperitas*	16	0	0
	Rhinocochlis	0	1	0
	Sasakina	4	0	0
	Elaphroconcha	10	0	0
	Kalamantania	1	0	0
	Everettia	16	0	0
	Quantula	1	0	0
	Bertia	0	1	0
	Dyakia	0	20	0
	Pseudoplecta	1	0	0
	Inozonites	10	0	0
Enidae	*Coccoderma*	5	0	0
Plectopylidae	*Chersaecia*	2	18	0
	Endoplon	2	0	0
	Plectopylis	0	6	0
	Endothyrella	2	0	0

Table S3. Records of living *Pareas carinatus* in the wild.

Forest type	Habitat	Height above the ground (m)	Year	Locality	Province	Source
Monsoon evergreen forest	Tree	1.0	2007	Khao Kho Hong	Songkhla	Arpapan Prakobkarn
Low land forest	Tree	2.0	2008	Kang Hang Maew	Chanthaburi	Patchara Danaisawadi
Teak plantation	Tree	1.7	2009	Doi Chiang Dao	Chiang Mai	Chirasak Sutcharit
Low land forest	Tree	1.0	2010	Kang Hang Maew	Chanthaburi	Patchara Danaisawadi
Low land forest	Tree	1.4	2010	Kang Hang Maew	Chanthaburi	Patchara Danaisawadi
Low land forest	Tree	1.5	2010	Kang Hang Maew	Chanthaburi	Patchara Danaisawadi
Low land forest	Tree	1.8	2011	Phliu Waterfall	Chanthaburi	Yumi Nakadera
Low land forest	Tree	1.5	2011	Kang Hang Maew	Chanthaburi	Patchara Danaisawadi
Low land forest	Vine	1.8	2011	Kang Hang Maew	Chanthaburi	Patchara Danaisawadi
Low land forest	Tree	1.5	2011	Kang Hang Maew	Chanthaburi	Patchara Danaisawadi
Low land forest	Vine	2.0	2011	Kang Hang Maew	Chanthaburi	Patchara Danaisawadi
Low land forest	Tree	1.4	2011	Phliu Waterfall	Chanthaburi	Chattraphas Pongcharoen
Monsoon evergreen forest	Tree	0.5	2012	Kuraburi	Phang Nga	Chirasak Sutcharit
